# Dietary behaviour and physical activity policies in Europe: learnings from the Policy Evaluation Network (PEN)

**DOI:** 10.1093/eurpub/ckac148

**Published:** 2022-11-29

**Authors:** Wolfgang Ahrens, Hermann Brenner, Marion Flechtner-Mors, Janas M Harrington, Antje Hebestreit, Carlijn B M Kamphuis, Liam Kelly, Michael Laxy, Aleksandra Luszczynska, Mario Mazzocchi, Celine Murrin, Maartje P Poelman, Ingrid Steenhuis, Gun Roos, Jürgen M Steinacker, Frank van Lenthe, Hajo Zeeb, Joanna Zukowska, Jeroen Lakerveld, Catherine B Woods

**Affiliations:** Leibniz Institute for Prevention Research and Epidemiology – BIPS, Bremen, Germany; Institute of Statistics, University of Bremen, Bremen, Germany; Division of Clinical Epidemiology and Aging Research, German Cancer Research Center (DKFZ), Heidelberg, Germany; Division of Sports and Rehabilitation Medicine, Department of Medicine, University Hospital Ulm, Ulm, Germany; HRB Centre for Health and Diet Research, School of Public Health, University College Cork, Cork, Ireland; Leibniz Institute for Prevention Research and Epidemiology – BIPS, Bremen, Germany; Department of Interdisciplinary Social Science, Utrecht University, Utrecht, the Netherlands; Department of Physical Education and Sport Sciences, Faculty of Education and Health Sciences, Physical Activity for Health Research Cluster, Health Research Institute, University of Limerick, Limerick, Ireland; Technical University of Munich, Professorship of Public Health and Prevention, Munich, Germany; SWPS University of Social Sciences & Humanities, Wroclaw, Poland; Department of Statistical Sciences, University of Bologna, Bologna, Italy; School of Public Health, Physiotherapy and Sports Science, University College Dublin, Belfield, Dublin, Ireland; Chair Group Consumption and Healthy Lifestyles, Wageningen University & Research, Wageningen, The Netherlands; Department of Health Sciences, Faculty of Science and Amsterdam Public Health Research Institute De Boelelaan 1085, VU University Amsterdam, Amsterdam, The Netherlands; Consumption Research Norway, Oslo Metropolitan University, Oslo, Norway; Division of Sports and Rehabilitation Medicine, Department of Medicine, University Hospital Ulm, Ulm, Germany; Department of Public Health, Erasmus MC, University Medical Center Rotterdam, Rotterdam, the Netherlands; Department of Human Geography and Public Health, Utrecht University, Utrecht, The Netherlands; Leibniz Institute for Prevention Research and Epidemiology – BIPS, Bremen, Germany; Health Sciences Bremen, University of Bremen, Bremen, Germany; Faculty of Civil and Environmental Engineering, Gdansk University of Technology, Gdansk, Poland; Department of Epidemiology and Biostatistics, Amsterdam Public Health, Amsterdam UMC, Vrije Universiteit Amsterdam, Amsterdam, the Netherlands; Upstream Team, Amsterdam, the Netherlands; Department of Physical Education and Sport Sciences, Faculty of Education and Health Sciences, Physical Activity for Health Research Cluster, Health Research Institute, University of Limerick, Limerick, Ireland

## Abstract

The European Policy Evaluation Network (PEN), initiated in autumn 2018, aimed at advancing the evidence base for public policies impacting dietary behaviour, physical activity and sedentary behaviours in Europe. This is needed because non-communicable diseases—the leading cause of global mortality—are substantially caused by physical inactivity and unhealthy dietary behaviours, which in turn are driven by upstream factors that have not yet been addressed effectively by prevention approaches. Thus, successful policy interventions are required that target entire populations and tackle the ‘causes of the causes’. To advance our knowledge on the effective implementation of policies and their impact in terms of improving health behaviours, PEN focused on five research tasks: (i) Adaptation and implementation of a Food Environment Policy Index (Food-EPI) and development of a Physical Activity Environment Policy Index (PA-EPI); (ii) Mapping of health-related indicators needed for policy evaluation and facilitating a harmonized pan-European approach for surveillance to assess the impact of policy interventions; (iii) Refining quantitative methods to evaluate the impact of public policies; (iv) Identifying key barriers and facilitators of implementation of policies; and (v) Advance understanding the equity impact of the development, implementation and evaluation of policies aimed at promoting physical activity and a healthy diet. Finally, and in order to provide concrete evidence for policymaking, existing exemplary policies, namely sugar-sweetened beverages taxation, active transport policies and school policies on nutrition and physical activity were assessed in consideration of these five tasks. At the end of the PEN project’s formal runtime, considerable advancements have been made. Here, we present an overview of the most important learnings and outputs.

## Introduction

To improve public health and to prevent non-communicable diseases (NCDs), the World Health Organisation (WHO) recommends a healthy diet, regular physical activity and limitation of sedentary behaviour. Despite all the evidence of benefits, epidemiological data indicate that few children, adults and older adults meet the physical activity or healthy dietary guidelines and suffer from poor nutrition and high levels of sedentary behaviour. There is growing consensus that a multi-level response that addresses personal, environmental and policy factors is needed.^1^ Approaches that address determinants of health behaviours at multiple levels have been used to successfully reduce the consumption of tobacco products.^2^ In parallel, there is a need for policies targeting the upstream determinants of health behaviours in an effort to reduce the immense burden of lifestyle-related NCDs. This calls for a systems-based approach that addresses the systemic drivers of inactivity and poor diet. The role of policy is to change systems instead of focussing on individuals, and in doing so, create supportive contexts in which programmes and environments collectively can reduce NCDs, including obesity. We consider public policies as purposeful decisions, plans and actions made by governments in a system designed to create system-level change to achieve specific societal goals. These are usually expressed in a law, a regulation or an order.

**Figure 1:**
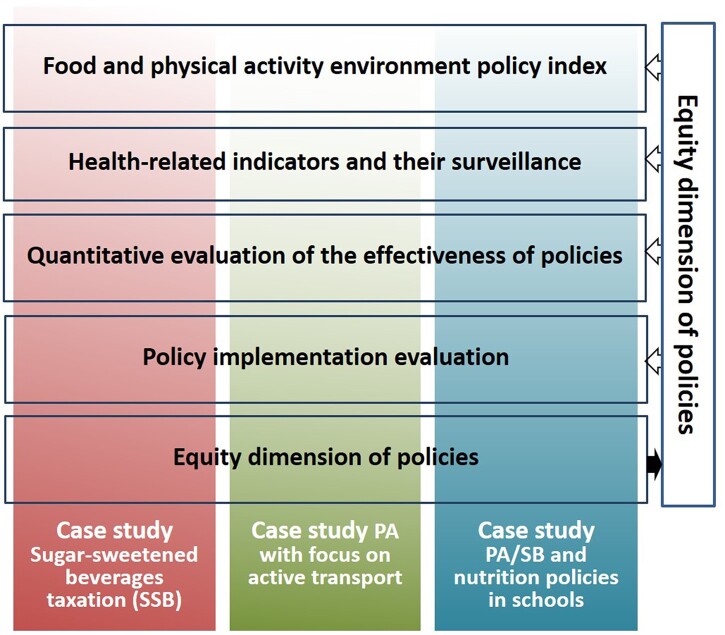
Overview of PEN’s major research areas and their mutual linkages

Despite the importance of understanding how policies can improve population diets and physical activity levels, until recently, there was hardly any knowledge on the implementation and evaluation of policy interventions across Europe. The European Policy Evaluation Network (PEN) was initiated to advance the evidence base for public policies impacting dietary behaviour, physical activity and sedentary behaviour in Europe.^3^ In particular, PEN linked 27 research institutes from various disciplines across eight countries who jointly have (i) assessed public policies with potential influence on food and physical activity environments, (ii) fostered progress towards a harmonized pan-European monitoring and surveillance system, (iii) modelled the impact of policies at the population level, (iv) evaluated policy implementation processes and their facilitators and barriers, and (v) gave recommendations for an equity and diversity perspective in policy development and evaluation (figure 1). With its multi-disciplinary network for the monitoring, benchmarking and evaluation of policies that affect dietary and physical activity as well as sedentary behaviour, PEN contributed to the improvement of population health. These activities were guided by a framework that helped us understand the concepts that inform the policy process as it pertains to diet and physical activity. The overarching systems-based PEN framework visualizes the complex and dynamic interrelations between policy domains, i.e. policy development, policy implementation and policy outcomes, the influence of contextual factors, and the importance of an equity dimension in designing and implementing such policies.^4^ The present paper provides a brief overview of the learnings of PEN and the evidence it obtained in regard to European policies related to diet, physical activity and sedentary behaviour.

The overview of PEN’s major scientific advancements is structured by the research areas as described above and as depicted in figure 1. While many studies are currently still underway, we summarize and paraphrase parts of the finalized and published work, and refer to more in-depth information where appropriate.

## Public policies with potential influence on food and physical activity environments

### Benchmarking of food policies

It has been widely acknowledged that unhealthy food environments are a driver of poor population diets and obesity. Moreover, food environments have been identified ‘as the interface where people interact with the wider food system to acquire and consume foods’.^5^ From a population health perspective, the current food system is failing, pushing food quantity over quality. This system is not supportive in helping consumers to make healthy food choices in line with recommended nutrition outcomes.^6^ Governmental policy and infrastructure support have the opportunity to improve food environments by implementing effective policies to ensure that the healthy food option is the default option and to empower citizens to make healthier choices and reduce levels of overweight, obesity and NCDs by creating supportive food environments.

We investigated country-level policies (in the Netherlands, Ireland, Norway, Germany and Poland) and European Union (EU)-level policies affecting food environments.^7–10^ Guided by the INFORMAS approach.^11^^,^^12^ PEN explored the development and use of a policy benchmarking tool called ‘the Food Environment Policy Index’ (Food-EPI) to improve our understanding of healthy food environments. The results of the Food-EPI studies across five European countries are compared, showing differences and similarities in achievement of healthy food environments. More specifically, PEN (i) compared the implementation of policies and infrastructure support and identified the most common implementation gaps in the mentioned five countries and the EU and (ii) identified the most prioritized policy and infrastructure support actions (based on importance, achievability and equity) in these countries to create healthy food environments in the EU.

PEN researchers selected key indicators for a healthy food environment, compiled international benchmarks for each indicator and collected evidence of the policy status in the selected countries and EU. A comprehensive list of the international benchmarks can be found in the country-specific evidence documents available on the PEN website.^13^ Expert panels identified and prioritized actions needed to address critical gaps in government policies and infrastructure support and reduce the rates of NCDs in Europe, with respect to health inequalities. Progressive, evidence-based and equitable food policies must be adopted to tackle the unhealthy and unequal food environments in Europe. Therefore, and for the first time, PEN incorporated an assessment of socio-economic inequalities into the Food-EPI tool. A concerted effort by policymakers to develop robust policies is necessary to reverse the trend of deterioration of our food environments and move to establishing healthier food environments for all. Concretely, the following conclusions were drawn from the work:

1. In all countries, the level of implementation of infrastructure support actions was higher than the implementation of policies directly shaping food environments. Also in the EU, infrastructure support action was stronger.
2. With the exception of Norway, all countries under study had predominantly ‘low’ to ‘very low’ implementation scores for policies directly shaping food environments.
3. The proposed priority actions to improve food environments in all countries and the EU were distributed over multiple sub-components of the policy and infrastructure support domains. They clearly outline the need of a comprehensive policy package covering multiple areas to improve food environments and public health nutrition.
4. Shared priority action areas across the five countries and the EU included:
  - Food price policies to make unhealthy foods more expensive and healthy foods less expensive;
  - Setting nutrition standards in public policies;
  - Regulation of food marketing to children;
  - The need for strong leadership.
5. Not all countries proposed actions where implementation gaps were highlighted.
6. Allocating resources to health promotion and disease prevention, as well as adequate monitoring of these efforts and improvements of the food environment, are essential.

### Benchmarking of policies targeting physical activity

Despite the fact that the UN Sustainable Development Goals and the WHO Global Action Plan on Physical Activity (WHO-GAPPA^14^) both highlight the need to move beyond *individual* behaviour change to broader *policy and system* approaches,^15^ the systematic evaluation, benchmarking and continuous monitoring of public policies to promote physical activity is in its infancy and remains a challenge both from an academic and a practical perspective. This is urgently needed as insufficient physical activity is a global issue for health. A multi-faceted response, including government action, is essential to improve population levels of physical activity.^16^ An unhealthy physical activity environment may be caused by a lack of ‘upstream’ policy progress in domains known to have a positive impact on physical activity behaviour, and when combined with a lack of effective infrastructure support for policy implementation, then the inactivity pandemic is likely to sustain, as the ‘system’ or environment remains unchanged despite best ‘downstream’ or programmatic efforts.^17^

Thus, PEN has developed a Physical Activity Environment Policy Index (PA-EPI) to assess the extent to which national government policies and actions towards creating a healthy physical activity policy environment have been implemented. The PA-EPI monitoring framework builds on learnings from the INFORMAS Food-EPI, adapted to answer ‘How much progress have governments made towards good practice in improving the physical activity environment and implementing physical inactivity/NCD prevention policies and actions?’ The PA-EPI is the first attempt at developing a tool that aims to assess the extent of implementation of government policies and actions, with the goal of creating a policy index to assess the healthiness of the physical activity environment.

**Figure 2:**
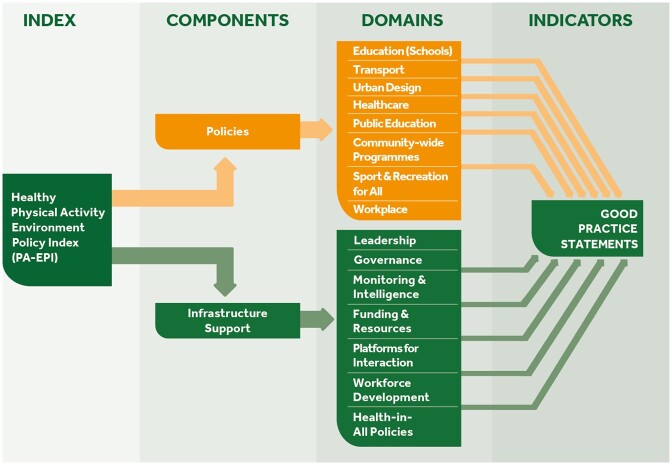
The Physical Activity Environment Policy Index (PA-EPI) Framework

The PA-EPI (figure 2) is conceptualized as a two-component ‘Policy’ and ‘Infrastructure Support’ framework which includes 15 domains, namely:

- **Policy domains (*N* = 8):** education, transport, urban design, healthcare, public education (including mass media), sport-for-all, workplaces and community.
- **Infrastructure support domains (*N* = 7):** leadership, governance, monitoring and intelligence, funding and resources, platforms for interaction, workforce development and health-in-all-policies.

We conducted a comprehensive consultation with academic experts and policymakers using both quantitative and qualitative data, alongside theoretical and pragmatic considerations, to inform PA-EPI development. Overall, 52 potential good practice statements across both the policy and the infrastructure domains were assessed regarding their relevance and clarity and rated according to their ‘importance’ in addressing population inactivity, their ‘feasibility’ to implement and their ‘ease of assessment’ in the online Delphi process. This resulted in 45 ‘good practice statements’ or indicators of ideal good practice across the domains listed above (figure 2).

So far, the PA-EPI tool offers the following perspectives:

- It may be used to independently monitor and benchmark public sector physical activity policies and actions.
- It can help identify and prioritize actions needed to address critical gaps in government policies and infrastructure support for implementation.
- It should evolve into benchmarks established by governments at the forefront of creating and implementing policies to counteract physical inactivity. Thus, providing pathways to help advise countries on how they may reach their individual goals and targets.
- The PA-EPI framework, its good practice statements and resources for implementation can be found on the PEN website (www.jpi-pen.eu).

### Providing the data basis to assess the effectiveness of policy measures—surveillance

The evaluation of the effectiveness of population-level policies with regards to behaviour and health outcomes requires methodological approaches other than randomized controlled trials. Temporal changes and regional differences of such outcomes in response to policy action can be assessed and monitored over time by systematic data collection and regular representative population surveys assessing key indicators that reflect these outcomes. These indicators must be suitable to identify specific and measurable characteristics of changes that demonstrate progress towards policy outcome or impact. Indicators should capture whether a policy has made changes to one or more levels of influence (policy, environment, organization, community, household, individual).^18^ It is a particular challenge to measure upstream indicators for dietary behaviour and physical activity. Therefore, a consolidated approach is needed to provide comparable health indicators across European surveillance and monitoring systems which can measure the evidence of change in response to the implementation of certain policies. PEN partners from Germany, Ireland, Poland, Norway and the Netherlands successfully took first steps towards a gradual harmonization across existing surveillance and monitoring systems covering different age groups, such as WHO Childhood Obesity Surveillance Initiative (WHO COSI; children), WHO Health Behaviour in School-aged Children (WHO HBSC; adolescents), WHO STEPwise approach to NCD risk factor surveillance (WHO STEPS; adult population), European Health Interview Survey (EHIS; adult population), and Nordic Monitoring of Diet, Physical Activity and Overweight (NORMO; young and adult populations).

Key indicators previously identified and prioritized by PEN and relevant experts^19^ were mapped against variables from 17 ongoing European monitoring and surveillance systems.^20^ These systems are run by 12 different organizations, such as Eurostat, the European Commission, or the WHO Regional Office for Europe. We derived a catalogue of key policy indicators from the corresponding monitoring and surveillance datasets that includes 72 indicators on multiple levels for dietary behaviour and 67 for physical activity. Several data gaps in current European monitoring and surveillance systems became apparent:

- Only few systems measure the upstream indicators, such as policy and contextual indicators together with the individual indicators (determinants and behaviour/health).
- Missing upstream indicators include those related to inequality, retail environment, and funding and resources supporting healthy dietary behaviour or physical activity.

The catalogue can be accessed on the PEN website.^21^ Stakeholders may use the policy indicator catalogue to identify indicators that can inform policy development and improve evaluation of corresponding policies. Filling the gaps regarding upstream indicators of the determinants of dietary behaviours and physical activity may facilitate a systems-level approach to policy development and evaluation. Policymakers may require cooperation between surveillance and monitoring systems to harmonize the indicators assessed for comparisons between populations across national borders.

**Figure 3:**
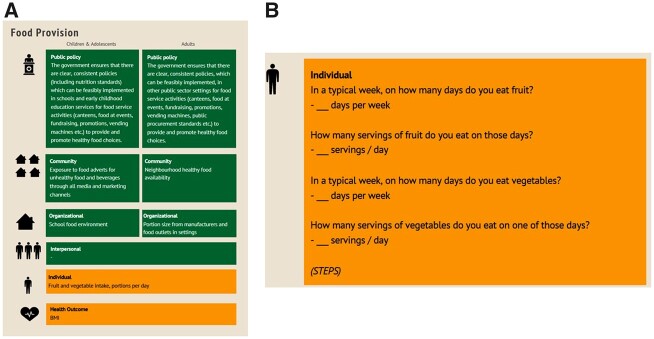
(a) SIMPLE modules, example for ‘Food Provision’: Indicators at systemic and individual levels. (b) SIMPLE modules, reference for data source

To assess and increase the comparability of data across surveys, age groups, and countries, measurement instruments for pan-European key health indicators were selected according to their scope, robustness and validity. Modular short questionnaire items that can be easily added to the systems’ mandatory (core) questionnaires were selected from the established questionnaires of ongoing surveillance initiatives. We used information from the policy indicator catalogue to initiate the harmonization process to develop such unified questionnaire modules, the Selected Instruments for Multilevel PoLicy and impact Evaluation (SIMPLE) modules.^22^ An example of behaviour and health indicators, assessed at different levels is depicted in figure 3a (systemic-level indicators in green, individual-level indicators in orange). Individual-level modules are considered as the starting point for the harmonization process and surveillance systems may successively integrate one or more of them in their core questionnaires. The newly added individual-level indicators can then be aligned with systemic level data (policy, community and organizational levels) that are routinely measured and included in existing monitoring datasets. The example in figure 3b shows WHO STEPS as a data source for fruit and vegetable intake.^23^ The modules can be accessed on the PEN website The modules can be accessed on the PEN website.^24^

We discussed the feasibility of implementing the SIMPLE modules as well as their usefulness for the evaluation of WHO and European health recommendations with representatives of the most important international and regional surveillance systems and they were supportive of harmonizing the assessment of key indicators. The barriers against implementing screeners were: the need to assess time trends; the limited space for additional questions; the required approval by authorities. The key facilitator was the need, by many systems, to update their instruments, for instance, by including sustainability indicators r better instruments to measure physical activity.

A protocol was developed for the establishment of a pan-European monitoring and surveillance system (i) allowing measurement of comparable public health data across systems, age groups and countries and (ii) facilitating the evaluation of change in policy-related outcomes across Europe.

### Modelling the (potential) impact of policies

The estimation of policy impacts and the assessment of the real-world and cost-effectiveness of population-based strategies to improve nutrition and physical activity behaviours is challenging because policy measures are hardly approachable by randomized controlled trials.^25^ To provide an empirical basis, quasi-experimental methods (QEM) using observational data are an alternative approach for policy evaluation. Offering a quantitative toolbox, QEM is successfully applied in the social sciences, while its application to identify causal effects of nutrition and physical activity policies on health outcomes is complex and not yet fully exploited.^26^^,^^27^ The association between policy and behaviour is probabilistic, lagged over longer time periods and not all data required may be available, particularly regarding confounding factors. Therefore, QEM cannot provide evidence on the long-term impact of policies on health outcomes.^28^ Consequently, mathematical disease simulation models (SM) projecting the long-term health and economic consequences are increasingly used.^29^

We have reviewed the strengths and limitations of different methodologies for the evaluation of policies targeting nutrition and physical activity behaviours, as well as their underlying general methodological assumptions. We considered laboratory and field experiments for the ex-ante assessment of policy impact through experimental methods, with an empirical case study comparing ‘*in vitro*’ and an ‘*in vivo*’ experiments to assess the impact of alternative nutritional labelling strategies in France.^30^ We produced a critical review on the application of QEM to evaluate the impact of nutritional policy with observational data. The methods were explored with two applications: the Catalunya soft drink tax, and the Cycling May campaign in Gdansk.^31^ We also developed and applied an empirical framework for the evaluation of the indirect effects of policies to simulate the consequences of agricultural and/or trade policies reform affecting the cost of raw sugar on prices and consumption of sugar-sweetened beverages (SSBs) in Italy.^32^ Finally, we simulated the effect of vitamin D food fortification and/or supplementation on cancer mortality in Europe.^33^

Based on this work, we draw a selected list of key implications/recommendations for impact evaluation. QEM and SM have strengths and limitations as standalone frameworks to estimate the impact of nutrition and physical activity policies. Thus, we propose to synergistically combine both approaches to overcome their limitations.^34^ However, assumptions behind models must be transparent and credible. This implies rigorous testing and validation through recognized robustness/sensitivity checks. Nutrition and physical activity policies may impact rapidly on behaviours, but the ultimate health effects may be delayed and only become apparent in the longer term.

### Effectively implementing physical activity and nutrition policies

**Figure 4:**
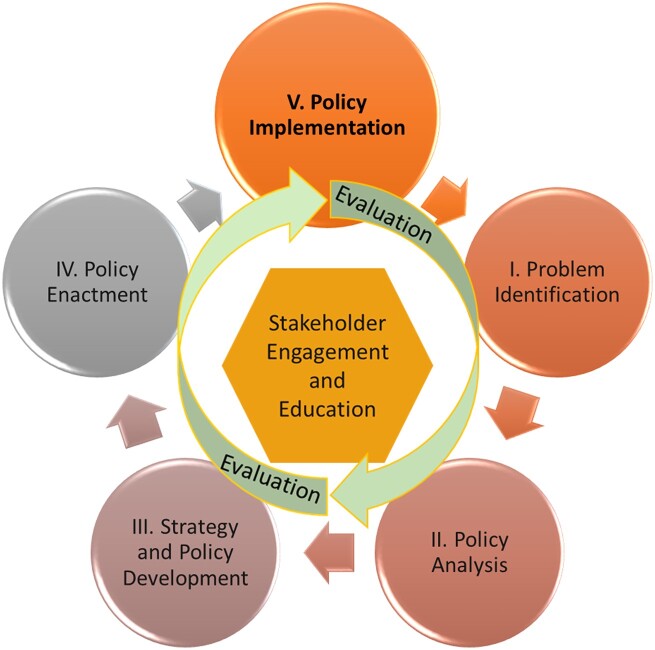
Policy Cycle: Adapted from: Centers for Disease Control and Prevention, Office of the Associate Director for Policy and Strategy^36^

Public policy interventions are usually laid down in policy documents that describe broad strategies, action plans, official guidelines or rules and legislation. However, their impact is determined by the degree corresponding specific actions are put into place by governmental or associated agencies to achieve a given public health objective. Thus, policy implementation and enactment are critical stages of the policy cycle (figure 4) where decisions are transferred into practice. Policy implementation evaluation is the assessment of ‘how’ the policy was put into practice. It can have multiple aims, such as: identifying determinants of implementation or its key processes, identifying differences between intended and actual implementation.^35^

The implementation of a policy can be considered successful if it creates a supportive context to reduce health risks and empowers individuals to adopt and maintain healthy behaviours. Implementation outcomes (e.g. acceptability of the way the policy is implemented) can be described as changes relating to the implementation process and are indicators for success,^37^ whereby factors that can influence these outcomes are referred to as implementation determinants, or barriers and facilitators.^38^ When conducting policy implementation evaluation, evaluation principles and methods should be applied that go beyond the concepts of ‘success’ or ‘failure’ and take into account policy actors, organizations, institutions and subsystems.^39^

We investigated frameworks guiding implementation of policies aiming at healthy nutrition, physical activity promotion and a reduction of sedentary behaviour. In particular, we aimed at examining the scope of the frameworks and the content of included constructs (e.g. referring to implementation processes, determinants or implementation evaluation), the level at which these constructs operate (e.g. individual, organizational/community), relationships between the constructs, and the inclusion of equity factors. Our review showed that most frameworks have a complex scope. They combine sections that are purely descriptive with sections accounting for prescriptive and/or explanatory associations.^40^ The majority of policy implementation frameworks have two or three aims combining process, determinants and/or evaluation of implementation, include multi-level constructs where system-level determinants are less frequently included than those from the individual or organizational/community level. Yet, frameworks include only few or no equity constructs.

Available systematic reviews indicate that implementation processes of policies promoting healthy dietary and physical activity behaviours are determined by various barriers and facilitators. However, an overarching synthesis of such reviews was missing. Thus, we conducted a meta-review of reviews and documents of major international stakeholders (e.g. from the WHO, the CDC), applying the Consolidated Framework for Implementation Research (CFIR). This was done to (i) identify determinants that were systematically indicated as occurring during the implementation processes and (ii) identify differences in the presence of determinants across reviews versus stakeholder documents regarding policies on these health-related behaviours targeting any population as well as those focusing on school settings. Across the 26 CFIR-based implementation determinants, seven were supported by 66.7–76.2% of reviews/stakeholder documents, namely cost, networking with other organizations/communities, external policies, structural characteristics of the setting, implementation climate, readiness for implementation and knowledge/beliefs of involved individuals. Most frequently, published reviews provided support for inner setting and individual determinants, whereas stakeholder documents supported outer and inner setting implementation determinants. Comparisons between policies promoting healthy diet with policies addressing physical activity or sedentary behaviour revealed shared support for only three implementation determinants: cost, implementation climate and knowledge/beliefs. In the case of policies targeting school settings, 14 out of 26 CFIR-based implementation determinants were strongly supported. The strongly supported implementation determinants may guide policymakers and researchers who need to prioritize potential implementation determinants when planning and monitoring the implementation of respective policies. The determinants cost, networking external policies, structural characteristics, implementation climate, readiness for implementation and knowledge/beliefs may be prioritized in the implementation processes.^41^

We conducted further meta- and systematic reviews that showed that (i) socio-cultural, economic and political contexts play a crucial role in successful policy implementation^42^^,^^43^ and that (ii) highly intrusive measures like taxation or restrictions are least likely to be accepted when first implemented, but confidence in relevance and effectiveness increases over time, which in turn may increase acceptance.^44^ We conducted scoping reviews on implementation processes of SSB taxation and physical activity policies to explore what happens after their enactment until possible effects are observed. Due to the small number of available studies, these two scoping maps did not allow firm conclusions about processes.^45^ It seems, however, that implementation of active design guidelines in planning and whole-of-city approaches are characterized by a coordinative approach, whereby implementation of national physical activity guidelines is a mix of different processes.

So far, the following recommendations on the implementation of policies can be given:

- When planning the evaluation of policy implementation, guidelines should be adhered to, such as the 10 steps defined by Ontario Agency for Health Protection and Promotion.^46^
- The use of implementation frameworks may help to address processes, determinants and evaluation of implementation while taking the interplay between contextual and equity factors into account.
- Stakeholders should get involved across the stages of policy implementation and its evaluation. The selection of relevant stakeholders from different groups and levels should be informed by the nature of the policy and the context in which it is implemented.

### Ensuring equity in health-related policies with a focus on food environments

Lower socioeconomic groups and ethnic minorities often have poorer health and less favourable health-related behaviours, including unhealthy food intake and lower levels of physical activity.^47^^,^^48^ Inequalities in dietary outcomes may partly stem from a higher exposure, an increased vulnerability to adverse food environments or both among lower socioeconomic groups. A PEN umbrella review found that food taxation may reduce socioeconomic inequalities in diets, but also that evidence for other food environment-related policies is poor.^49^

Importantly, we noted that lower and higher socioeconomic groups not only differ in the healthfulness of their dietary intakes but also in the material and sociocultural circumstances in which they are born, grow up, work and age, i.e. their daily living conditions. We illustrated how theories aimed at understanding socioeconomic inequalities in health, in which connections are made with daily living conditions may provide insights in the ultimate causes of socioeconomic inequalities in diets, and how these may affect the impact of food environment policies.^50^

There is a growing consensus that we can conceptualize health inequalities, the obesity epidemic, and therefore also inequalities in dietary intake as an ‘emergent property of a complex adaptive system’. We demonstrated how poor dietary intake in low-income groups can be considered as an emergent property of a complex adaptive system that sustains a food environment that tends to perpetuate increasing accessibility, availability, affordability and acceptability of unhealthy foods. Multiple interconnected feedback loops seem to shape an adverse food environment in these groups. The economic basis of the system results in a ubiquitous supply of energy-rich, nutrient-poor and ultra-processed foods, fuelling the demand for these products based on their social and cultural significance, availability and affordability.

We conclude that a systems-based model can help to identify ‘leverage points’ that may contribute to a change in the system; it is unlikely that a sustainable improvement of dietary intake can be achieved through isolated interventions. Instead, several strategies are needed simultaneously to facilitate longer-term management of household finances and socially oriented practices around healthy food production, supply and intake, in a system paradigm which gives more priority to health.

### Creating evidence for effective policies—methodological challenges and three applied examples

Quantifying the impact and cost-effectiveness of policies is challenging. Relative to clinical interventions, public policies are hard to randomize and it might take years or decades until the policies will translate in positive health outcomes. Both, quasi-experimental analysis approaches and simulation modelling are techniques to overcome these challenges. Within PEN we conducted an integrative review on the methods, challenges and potential synergies of both approaches for the evaluation of nutrition and physical activity policies.^34^ The application of these methods for policy evaluation increased substantially over the last decade. It is important that the assumptions of quasi-experimental and simulation modelling methods should be credible and rigorously tested and transparently communicated. Both approaches can and should be applied synergistically to improve the robustness of policy evaluation results.

The various research areas covered in PEN (and described above) are, in practice, often linked and relevant in combination rather than in isolation. We therefore showcased how specific policies can be evaluated regarding implementation and impact, and how the experience/knowledge gained across the research areas can be applied. Thus, we selected and assessed existing exemplary policies, namely SSBs taxation, active transport policies and school policies on nutrition and physical activity.

#### The case of the SSBs tax

It is estimated that 30–70% of the adult European population is overweight of which 10–30% are obese.^51^ Consumption of SSBs is considered as an important modifiable risk factor for overweight and obesity.^52^ The decrease in purchases and consumption of SSB seems proportional to the tax rate applied.^53^ Modelling studies indicate that if the tax rate is 20% or more, SSB taxes have the potential to reduce the prevalence and incidence of overweight and obesity, diabetes type 2, dental caries and to reduce disability-adjusted life years.^54^ However, the acceptability of government interventions perceived as intrusive, such as an SSB tax, is suggested to be generally low.^55^ It is therefore not surprising that countries have experienced challenges in the introduction of an SSB tax due to opposition of the beverage industry and low political and public acceptability.^54^^,^^56^ Still, an SSB tax is regarded as the most feasible health-related food tax to implement.^57^ SSBs are regarded as cost-effective from a public policy point of view, as their implementation costs are low and they raise revenues that may be ringfenced to fund other health-promoting policy interventions.^58^ Currently, SSB taxes have been implemented in over 40 countries worldwide.^59^ An important equity aspect is the fact that an SSB tax is regressive—i.e. the cost burden falls disproportionally on lower socioeconomic groups. However, an SSB tax also seems to have progressive health effects—i.e. the beneficial health effects are most pronounced in lower socioeconomic groups, suggesting that an SSB may contribute to the reduction of health inequalities.^60^

**Figure 5:**
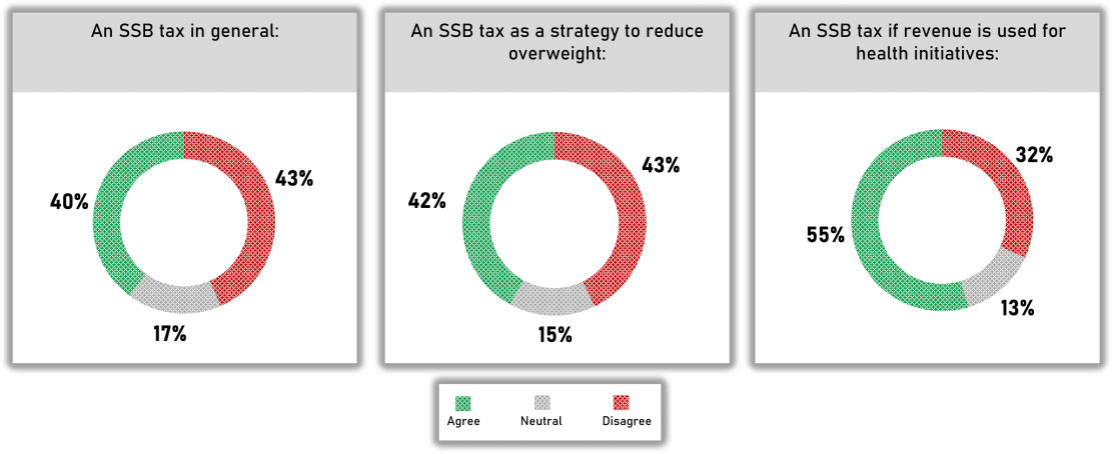
Public support for SSB tax in the Netherlands^61^

Our systematic review identified five important factors that affect the political and public acceptability of an SSB tax: (i) beliefs about effectiveness and cost-effectiveness, (ii) beliefs about appropriateness, (iii) beliefs about economic and socioeconomic benefit, (iv) beliefs about policy adoption and implementation and (v) public mistrust towards the industry, government and public health experts.^43^ Furthermore, we conducted an online survey including a sample of 500 Dutch adults (representative of the Dutch population for age, sex, educational level and location) to investigate public acceptability of an SSB and its associated factors in the Netherlands.^61^ Public acceptability of an SSB tax tends to be higher if revenue is used for health initiatives. Indeed, the findings indicate that in the Netherlands, the majority of the public (55%) supported an SSB tax if the revenue generated from the tax is used for health initiatives (figure 5).^61^ Acceptability was lowest in participants with a low educational level, overweight, moderate or high SSB consumption and those with adolescent household members.

A qualitative study among stakeholders showed that several obstacles impede the adoption of an SSB tax in the Netherlands—e.g. considerable disagreement among stakeholders, an unfavourable political context and a strong lobby (particularly from the food and beverage industry) against an SSB tax.^62^ Stakeholders agreed that an SSB tax would have a larger impact on the budgets of lower socioeconomic groups. But a tax could also have greater health benefits among those groups and thus reduce socioeconomic inequalities in dietary intake and health.^63^ To be most effective, additional interventions should be considered, for instance, decreasing the prices of healthy foods.

Our randomised controlled trial (RCT) using virtual supermarket software showed that an SSB tax could be effective in reducing SSB purchases.^64^ The World Bank recommends that taxes on SSBs raise retail prices by at least 20% to reduce consumption.^54^ The SSB tax introduced in Catalonia in May 2017 (corresponding to around 10% of the average price) proved to be effective in increasing prices but did not significantly affect drink purchases, according to our estimates using a quasi-experimental approach on data from the Spanish Household Budget Survey. The RCT showed that more beneficial effects on consumer food purchases could be expected from a nutrient profiling tax based on Nutri-Score targeting a wider range of foods and beverages with a low nutritional quality.^64^

In conclusion, the following recommendations can be made:

- Use the revenue generated from an SSB tax for health initiatives.
- Form advocacy coalitions to support the introduction of an SSB tax.
- When introducing an SSB tax, raise retail prices by at least 20% to reduce consumption.
- Look for opportunities to broaden the tax base.
- Couple an SSB tax to societal problems other than public health.
- Combine the introduction of an SSB tax by other interventions to reduce SSB consumption in lower socioeconomic groups.

#### The case of sustainable urban mobility plans

A shift towards transport modes enhancing physical activity, further called ‘active transport’ could contribute to meeting the WHO recommendations on physical activity.^65^ The European Commission has long supported the implementation of sustainable urban mobility plans (SUMPs^66^) as an approach to strategic and sustainable mobility planning. Public transport users may gain an additional 8–33 min of walking attributable to each trip.^67^ Walking and cycling, however, are often confined to neighbourhoods, might take more time and effort and cause inconvenience. Soft interventions such as educational programmes can be very effective and lead to a 7% reduction in car modal split share.^68^ Yet, the general impact of transport-related policies and interventions on physical activity levels is poorly understood. The strength of the evidence varies from very low to moderate.

We identified three main transport policy areas contributing to higher physical activity levels: (i) convenient transport infrastructure development, (ii) active transport promotion and (iii) shift of transport mode.^69^ Results of a meta-analysis indicate that active transport-related interventions significantly reduce car use.^70^ An evaluation of the effect of SUMP implementation on physical activity levels was restricted by data availability. Limited data availability and a variety of indicators impede a cross-city comparative evaluation of the effectiveness of SUMPs.^71^

**Figure 6:**
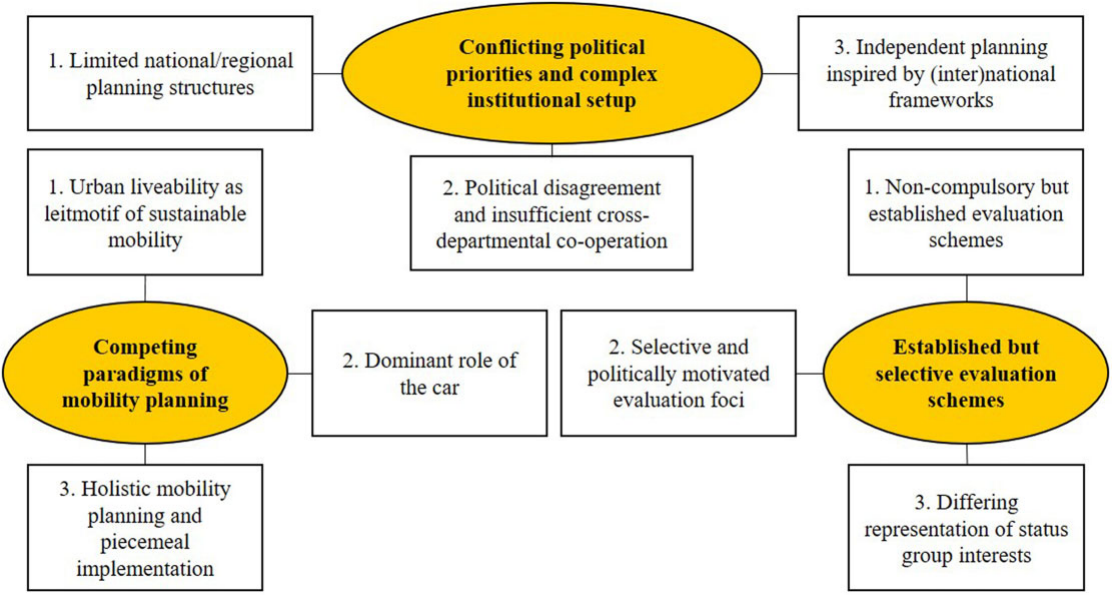
Thematic map for interviews on sustainable urban mobility plans implementation in Copenhagen, Denmark.^71^

Complex institutional structures, the dominant role of motorized traffic as well as complex regional and local policy integration hamper SUMP implementation. In cities advanced in SUMP implementation, mobility strategies are aligned with broader sustainability themes. Cities less experienced in active mobility could utilize a similar strategy. However, car-oriented planning is grounded in culture, nurtured by economic dependencies and perpetuated by hesitant policymakers.^71^ An example for the forces to be considered is illustrated in figure 6.

We conclude that policy interventions aimed at infrastructure development but also educational programmes and any indirect interventions with potential to achieve substantial shifts towards active transport are most promising. Physical activity levels can be increased by implementing policies that provide convenient, safe and connected walking and cycling infrastructures, promote active travel and support to public transport. There is also strong evidence that active travel policies work best when implemented comprehensively and coherently. This may include infrastructure and facility improvements as well as educational programmes to achieve substantial shifts towards active modes of travel. Level of motorization, modal split and public-transport use were identified as common and suitable indicators for monitoring changes in transport-related physical activity levels. Sufficient financial resources, horizontal and vertical cooperation between agencies as well as a fundamental emphasis on sustainable transitions are crucial for successful SUMP implementation.

#### The case of school-based fruit and vegetable provision programmes

A higher fruit and vegetable consumption is associated with lower risk of all-cause mortality, and evidence supports the recommendations of 400 g or five portions of fruit/vegetables per day. However, only 40% of European 7- to 9-year-olds and 11–13–15-year-olds eat fruit daily.^72^^,^^73^ The proportion of children who consume fruit and vegetables has remained unchanged for 20 years.^74^ To reverse this, better understanding of implementation and long-term impact of school-based fruit and vegetable provision programmes (SFPPs) is needed. The benefits of implementing such programmes outweigh the costs of not doing so assuming that 30% of the effect will be maintained over time.^75^

We conducted a systematic qualitative review of 14 studies reporting on the barriers and facilitators to implementation of interventions that entail the action of direct provision of fruit and vegetables in kindergarten and school settings.^76^ The CFIR was used and the following determinants in the implementation of fruit and vegetables interventions in schools were identified: (i) intervention characteristics domain: ‘design quality and packaging’, ‘adaptability’ and ‘cost’; (ii) outer setting: ‘cosmopolitanism’, external policy and incentives’ and ‘patients’ needs and resources’; (iii) inner setting: ‘implementation climate’, ‘readiness for implementation’ and ‘structural characteristics’; (iv) characteristics of individuals: ‘individual stage of change’, ‘knowledge and beliefs about the intervention’ and finally of (v) process: ‘engaging’, ‘executing’ and ‘reflecting and evaluating’. The review stresses the dual role of parents as both supporting the implementation and targets of the intervention. Positive child perceptions of the value of the intervention and perceived behaviour change due to the intervention were reported as relevant facilitators to implementation across several studies. We concluded that CFIR provides a systematic approach for identifying and organizing the implementation determinants of nutrition interventions in kindergartens and schools. Revisions are encouraged to provide adequate space for the perceptions of various implementation actors and the target group.

Furthermore, we conducted 23 semi-structured interviews with stakeholders from ministries of agriculture, health and education, across 10 EU member states and with a representative from the EU level. We aimed to understand barriers and facilitators to implementation of the European School Fruit and Vegetable Scheme (EU-Scheme) based on perceptions from those responsible at government level and consider the applicability of the CFIR for this purpose. The qualitative data were mapped to the domains/constructs/sub-constructs of the CFIR. Flexibility in how the EU-Scheme is designed and implemented enables country level implementation, and newly established cooperation between implementing ministries is a potential facilitator. However, timing of the top-down budget allocation is a barrier and taking EU funding for granted is a potential disincentive to improvement although the funding facilitates sustainability. Despite agreement on what the overall goals of the EU-Scheme are, there is some ambiguity as to what the primary goal is, which may influence design as well as implementation at country and school level. This ambiguity seems to translate into a potential barrier to design and implementation at country and school levels.

Finally, we applied a systems approach to provide an integrated perspective of the mechanisms of the EU-Scheme to understand better how to increase its long-term impact on children’s fruit and vegetables consumption.^77^ In online meetings with 10 experts in school-based fruit and vegetable programmes, children’s fruit and vegetable consumption, and the EU-Scheme causal loop diagrams were developed. The findings suggest that a central self-reinforcing mechanism through which children socialize during fruit and vegetables consumption is critical in the habituation process. In addition, the initial increase in children’s fruit and vegetables consumption following the EU-Scheme implementation is due to growth in three self-reinforcing loops related to motivation and capability mechanisms; however, this trend gradually slows and stops due to four balancing feedback loops with alternative goals related to opportunity mechanisms that reach their limits. We conclude that children’s fruit and vegetables consumption can be maintained over time when their motivation and capabilities are combined with sustained opportunities. Because multiple actors and settings influence children’s motivation, capability and opportunity, activities that can align them and their objectives should be included in the EU-Scheme.

Lack of time, adequate human resources and tools like teaching materials or websites linked to the interventions are important barriers to the implementation of SFPPs. Sustained financial resources that ensures frequent and free FV provision that reaches to as many children as possible can enhance the impact of the SFPPs. Our results support the following recommendations:

- Time and effort should be invested in establishing and cultivating the relationships between suppliers of fruit and vegetables and kindergartens/schools before and during the implementation.
- Teachers, children and those involved in distributing the fruit and vegetables should be consulted on appropriate design, packaging, as well as frequency of delivery and overall duration of the SFPPs throughout the school year.
- Nutrition-related policies in Europe/nationally should make use of the relationships between ministries of agricultural, health, and education established by the European School FV Scheme.
- SFPPs activities should align the actors and their objectives across settings to address children’s motivation and capabilities combined with sustained opportunities to eat fruit and vegetables.

## Next steps

### Training of early careers researchers

PEN established an Early Careers Network (PEN-ECN), a network of self-defined ‘early career’ researchers and practitioners to foster the interaction, capacity and career development in the field of dietary, physical activity and sedentary behaviour research at the population level. Program initiatives such as numerous webinars, a Journal and Coffee Club and a Mentorship Program have been successfully established and required regular and substantial commitment of participants. The governance document developed for the PEN-ECN will serve as a template to help define terms of reference, roles of members and to propose activities supporting career development. This has stimulated the formation of an ECN with early careers researchers from other JPI-HDHL projects with the ultimate aim to establish a sustainable overarching network of early careers researchers within the JPI-HDHL beyond the funding period of single projects.

### Application of the healthy Food-EPI

A final and important phase of the Food-EPI process involves the distribution of the recommendations to policymakers or the uptake of recommendations by public health organizations who advocate for policy change and infrastructure support to improve the food environment. The conduct of repeat Food-EPI assessments will facilitate the comparison of government actions between and within countries and measure their commitment to improving the European food environment. It is important to ensure accountability and maintain forward momentum despite changes in government leadership and other dynamic contextual factors. In the long-term, this research will contribute to a global database for monitoring and evaluating policies directed at improving the food environment and continuing obesity and NCD prevention commitments. Follow-up studies will be a key to demonstrating the development and strength of food environment policies occurring in the participating European countries. Monitoring progress in the implementation of food environment policies will contribute to the establishment of healthier food environments that enable healthier diets and reduce the burden of obesity and NCDs.

### Further development and implementation of the PA-EPI

It is anticipated that the PA-EPI tool will be further refined to enhance its usefulness for the next phase of research, which is benchmarking the level of implementation of policies to support and promote increasing population levels of physical activity. The first steps to apply the tool are currently taken in Ireland, and more countries will follow, depending on dedicated project funds. When funding is secured, conducting the PA-EPI would involve establishing a ‘national coalition’, a group of non-government public health and/or other stakeholders to manage the process or, alternatively, an existing public health non-governmental organistion (NGO) or association to take the lead.

### Improvement of surveillance systems

The proposed SIMPLE modules may serve as an entry point towards a harmonized European surveillance if they can be implemented successively in ongoing surveillance systems. They will assess health indicators in a comparable way, e.g. to evaluate a population's adherence to health recommendations and the impact of health policies. By initially piloting one or two modules, surveillance systems may enter into a stepwise up-scaling of modules and, ultimately, the implementation of more sophisticated instruments. Surveillance systems may introduce individual-level questionnaire items as supplementary modules or in sub-samples, or pilot the instruments in national surveys without discarding their established instruments. In future steps methodological studies investigating the validity and reliability of questionnaire items in different age-groups and evaluating their suitability to monitor WHO recommendations may be conducted. Further methodological studies may assess the introduction of digital technologies and wearables may replace parts of questionnaires, e.g. to assess physical activity. Such studies may be supported by a methodological competence platform with representation of different surveillance systems and stakeholders using surveillance data. This group may initiate and guide the further harmonization steps and methodological advancement of measurement instruments for surveillance.

### Better integration of evaluation methods with simulation modelling

Although methods based on experimental and observational data are powerful in identifying immediate behavioural effects, simulation models are a better tool to project the effect of behavioural changes on later health outcomes. The growing interest in personalized interventions, and the variability in the individual response to interventions call for the proper application of these methods to allow for variability in responses to policy, go beyond average policy effects, and consider the distribution of impacts across different population sub-groups. Multi-component lifestyle policies pose a major challenge in estimating the impact of individual measures. The joint application of QEM and SM has the potential to generate new evidence on multi-component policies.

### Effective implementation of policies

PEN has succeeded in strongly putting a lens on aspects of effective policy implementation and the evaluation of implementation actions. It has become clear that the use of evaluation guidelines and implementation frameworks is worthwhile and required to strengthen scientific rigour and transparency of implementation evaluation, and further advances in terms of promoting guideline and framework application should be aimed for, e.g. in the context of professional societies and training offers. To address the so far limited considerations of equity aspects and of stakeholder involvement in policy implementation evaluation, coalitions with experts in social epidemiology and complex systems perspectives need to be strengthened in concrete implementation activities. Guidance on stakeholder inclusion and involvement needs to be developed at the same time.

### Reduction of health inequalities

Improving dietary intake in lower socioeconomic groups for the purpose of reducing inequalities in dietary intake and physical inactivity remains a major challenge. PEN identified that taxation may reduce such inequalities; we also showed however, that more is needed. Essentially, connecting the development, implementation and evaluation to broader social conditions in life using a systems perspective may widen the policy options. While food environmental policies are pivotal, policies outside the domain of health may be contribute to the above-mentioned challenge. A system-based model can help identify interventions that have desirable knock-off effects on other parts of the system or to show how multiple interventions together can contribute to a change in the system. Co-creation with target groups and including a variety of stakeholders is recommended. Execution and delivery need to be sensitive to diversity of the target population in terms of affordability, accessibility and acceptability of policy actions. There is still much to be learnt, which needs to be done with stakeholders and target groups.

### Promising perspectives for future policy measures

The evaluations of the three policies that were investigated as case study in PEN, i.e. SSB taxation, active transport policies and school-based fruit and vegetable provision programs, show the potential of such policies for improving population health. Also, our evaluations show that the implementation of these policies is often complex, as policies are not implemented in a vacuum, but in a context where competing interests of various stakeholders, limited budgets, other policies and all kind of contextual factors affect implementation. The population health effects of policies should be assessed with various methods that complement each other (e.g. RCTs, natural experiments, simulation models), and the availability of reliable data to do so is an important priority.

## Data Availability

No new data were generated or analysed in support of this research.
